# A Biochemical Characterization of the DNA Binding Activity of the Response Regulator VicR from *Streptococcus mutans*

**DOI:** 10.1371/journal.pone.0108027

**Published:** 2014-09-17

**Authors:** Eduardo Ayala, Jennifer S. Downey, Lauren Mashburn-Warren, Dilani B. Senadheera, Dennis G. Cvitkovitch, Steven D. Goodman

**Affiliations:** 1 Department of Molecular and Computational Biology, Division of Biomedical Science, Herman Ostrow School of Dentistry, The University of Southern California, Los Angeles, California, United States of America; 2 Center for Microbial Pathogenesis, The Research Institute, Nationwide Children’s Hospital, Columbus, Ohio, United States of America; 3 Dental Research Institute, Faculty of Dentistry, University of Toronto, Toronto, Canada; LSU Health Sciences Center School of Dentistry, United States of America

## Abstract

Two-component systems (TCSs) are ubiquitous among bacteria and are among the most elegant and effective sensing systems in nature. They allow for efficient adaptive responses to rapidly changing environmental conditions. In this study, we investigated the biochemical characteristics of the *Streptococcus mutans* protein VicR, an essential response regulator that is part of the VicRK TCS. We dissected the DNA binding requirements of the recognition sequences for VicR in its phosphorylated and unphosphorylated forms. In doing so, we were able to make predictions for the expansion of the VicR regulon within *S. mutans*. With the ever increasing number of bacteria that are rapidly becoming resistant to even the antibiotics of last resort, TCSs such as the VicRK provide promising targets for a new class of antimicrobials.

## Introduction

Two-component signal transduction systems (TCSs) are used by bacteria to sense environmental cues and regulate gene expression in response to them [1]. TCSs are typically composed of a membrane-bound sensor histidine kinase (HK) [2] that perceives a signal and transmits it through phosphoryl transfer to a cognate cytoplasmic response regulator (RR) [3]. HKs are bifunctional proteins that can have both kinase and phosphatase activities [4]. Phosphorylation regulates the activity of the RRs which typically have DNA binding activity and serve as transcriptional regulators [5].

In *Streptococcus mutans* the VicRK TCS is essential and regulates key virulence attributes [6]. Genetic competence, biofilm formation, sucrose mediated adhesion, oxidative stress tolerance, acid production, acid tolerance, bacteriocin production and cell wall metabolism are all under the control of the VicRK TCS [6]–[11]. The conservation and essentiality across low G+C Gram-positive bacteria have made this TCS an attractive target for the development of new antimicrobials [12]. In fact, various inhibitors of VicK and VicR homologues in *B. subtilis, Staphylococcus aureus, and Staphylococcus epidermidis* have been identified and were shown to exhibit bactericidal effects on these organisms [13]–[15].

In *S. mutans*, the VicRK TCS is composed of the HK VicK and the RR VicR. A regulatory signal for VicK has not been identified conclusively though several are suspected, including cell wall precursors and oxidative stress [12], [16]. The crystal structure of the intracellular region of VicK was recently solved. It is composed of a signaling HAMP domain, a redox sensing PAS domain, a dimerization and conserved histidine DHp domain and a catalytic ATPase CA domain [17]. Wang *et al*. also confirmed the autokinase and phosphatase activities of VicK [17]. VicR is predicted to have a modular structure consisting of an N-terminal receiver domain that contains the conserved site of phosphorylation and a C-terminal DNA binding domain by the NCBI Conserved Domain Database (CDD) [18]. Based on sequence homology, VicR belongs to the OmpR family of RRs [19], whose members are characterized by their winged helix-turn-helix (wHTH) DNA binding motif, the recognition helix of which is able to bind to target nucleotides within the major groove of a DNA binding site [20].

The binding sequence for the VicR homolog in *B. subtilis* and *S. aureus*, WalR, has been characterized [21], [22]. The consensus sequence TGTWAH-N_5_-TGTWAH (where W is an A/T and H is an A/T/C) consists of two hexameric half sites separated by a non-conserved 5 base pair spacer. This sequence was used to successfully identify members of the WalR regulon in both *Bacillus* and *Staphylococcus*. The upstream regions of virulence genes (*gtfB*, *gtfC* and *ftf*) in *S. mutans* were analyzed previously and found to contain a match to the WalR consensus in their promoter regions [6].

VicR was shown to bind upstream of a number of virulence genes including *gtfB, gtfC*, *ftf, gbpB*, and *nlmC* by Electophoretic Mobility Shift Assay (EMSA) and footprinting [6], [7], [11]. VicR has also been shown by EMSA to bind upstream of *smu.367*, *smaA*, *wapE*, and *lysM* which are genes involved in cell envelope and cell wall biogenesis [23]. However with the exception of the *comC-nlmC* intergenic region binding study performed by us [11], no other groups have further delineated binding sites with *S. mutans* VicR.

In this study we performed DNaseI footprinting, EMSA and mutational analyses on known, suspected and novel members of the *S. mutans* VicR regulon and provide the largest footprinting survey and hence sequence determinants of the VicR regulon of *S. mutans* to date. As part of this survey we show for the first time the direct binding of VicR to *atlA*, *bmsH*, *glnQ*, *copY*, *wapA*, *relR*, *gcrR* and *plsX.* In addition, we performed a mutational analysis on the WalR consensus (TGTWAH-N_5_-TGTWAH) found upstream of *gcrR*. The *gcrR* site was chosen because it ranks as one of the highest affinity of the naturally occurring VicR sites and is a key regulatory gene of critical importance to the aciduric properties of *S. mutans*. This mutational analysis allowed us to refine the WalR consensus to one that reflects species-specific requirements of VicR binding in *S. mutans*.

## Materials and Methods

### VicR Phosphorylation with acetyl phosphate

Previously we had successfully performed EMSA experiments with an MBP-VicR [6]. During pilot binding experiments with the DNA substrate *gcrR* we found that a His-tagged version of VicR had greater binding affinity than the MBP-VicR (data not shown). Indeed MBP-VicR failed to produce DNase footprints on any DNA substrate tested (data not shown). This suggested that the MBP-tag potentially reduces the affinity of the VicR protein with DNA targets. Thus all experiments presented here use the smaller tagged His-VicR. Cloning and purification of VicR were performed as previously described [11]. Four µM His-VicR was incubated for 1 h 15 min at 37°C in phosphorylation buffer (50 mM Tris pH 7.4, 50 mM KCl, 2 mM MgCl_2_ and 20% glycerol) with 50 mM acetyl phosphate (AcP) in a reaction volume of 50 µL [17]. Seven µL of each phosphorylation reaction was mixed 1∶1 with SDS sample buffer (60 mM Tris HCL, 10% glycerol, 2% SDS) and loaded onto a Novex 4–20% SDS PAGE gel (Invitrogen). The gels were pre-chilled for a minimum of 3 h at 4°C and the proteins separated by electrophoresis at 4°C for 3 h at 10 Volts/cm. The phosphorylation status of VicR was observed as a reduction in mobility of phosphorylated VicR versus the unphosphorylated protein. Bands were visualized by silver stain [24]. For an unphosphorylated control, VicR was incubated in phosphorylation buffer minus the acetyl phosphate in a parallel reaction.

### Preparation of DNA substrates for footprinting

Labeled DNA substrates representing selected promoter regions of *atlA, bsmH, copY, fruA, gbpB, gcrR, glnQ, gtfB, gtfC, nlmC, plsX, relR,* and *wapA* were amplified by PCR using an isotopically labeled primer and an unlabeled primer (Table S1). Oligonucleotides were labeled using T4 polynucleotide kinase (New England Biolabs) and [γ-^32^P] ATP (PerkinElmer). With the exception of *gtfB*, the forward primer of each substrate was labeled. 2.5 units of T4 polynucleotide kinase were added to 2 µM of either the forward or reverse primer, 0.5 µM [γ-^32^P] ATP, and 1 X T4 polynucleotide kinase reaction buffer (70 mM Tris HCL, 10 mM MgCl_2_, 5 mM DTT pH 7.6) in a volume of 5 µl.

The plasmid pSUW1 [25], carrying the promoter and coding sequences of *gtfB* and *gtfC* from GS-5 *S. mutans*, was used as a template for *gtfB* and *gtfC* amplification. Presumed secondary structure in the promoter regions of these two genes prevented the use of chromosomal DNA as a template for PCR. PCR reactions for all other genes used chromosomal DNA from *S. mutans* UA159 as a template. The amplification reaction contained 1 X Go Tag Flexi buffer (Promega, proprietary formulation), 0.2 µM of each primer (Table S1), 0.2 mM dNTPs, DNA (chromosomal or plasmid), and 5 units of Go Tag Flexi polymerase (Promega) in a 50 µL reaction. The PCR reactions were incubated in a PTC-100 thermocycler (MJ Research) using a program with the following parameters: denaturing at 95°C for 1 min, annealing at 50°C for 1 min, extension at 72°C for 1 min. This was repeated for 30 cycles with a final extension step of 72°C for 15 min. PCR reactions were separated by electrophoresis on a 2% agarose gel and the target band was excised and purified using the Qiaquick Gel Extraction Kit (Qiagen). Non-isotopic substrates used in EMSA (*gcrR* and *gtfC*) were amplified as stated above with the kinase labeling step omitted. The concentration of the substrates was determined by UV spectrophotometric analysis [26] using a DU-640 UV-Vis Spectrophotometer (Beckman-Coulter).

### Preparation of *gcrR,* and *plsX* 77, 82 and 87 base pair DNA substrates for mutational analysis

Mutations in the putative VicR binding site within the promoters of *gcrR* and *plsX* were incorporated directly into oligonucleotides ordered from the University of Southern California Genomics Core. Complementary oligonucleotides representing wild-type and mutated versions of *gcrR* and *plsX* were used to generate a double stranded template for PCR (for primer sequences see Tables S2 and S3). This PCR product was then used as a substrate for EMSA and footprinting. The double-stranded substrates were generated as follows. Two partially complementary oligonucleotides were annealed to generate a double stranded substrate with single stranded 5′ regions. One oligo represented the 5′ end of the forward strand while the other represented the 5′ end of the reverse strand. The oligonucleotide pairs were complementary at their 3′ ends and each contained the putative binding site within the overlapping region. The oligonucleotides were allowed to anneal and extend for one cycle with Taq Polymerase. The extension reactions were incubated using a program with the following parameters: denaturing at 95°C for 1 min, annealing at 47°C for 1 min, extension at 72°C for 1 min followed by a second extension step at 72°C for 3 min.

An isotopically labeled forward primer and unlabeled reverse primer (Tables S2 and S3) were added once the extension reaction had completed and the newly synthesized double stranded templates were amplified by PCR in the same tube. Primers oSG712 and oSG713 (Table S2) were used to amplify all of the *gcrR* substrates for the mutational analysis. Primers oSG826 and oSG827 (Table S3) were used for *plsX* amplification. The PCR reaction was again carried out with the conditions described above. Amplified substrates were purified as was done in the previous section. Eight *gcrR* and four *plsX* variants were generated, each with the original or a mutagenized VicR binding site in the center of each substrate. The length of the mutant substrates are as follows: The +1 spacer mutant was 82 bp long, the +2 spacer mutant was 87 bp and the remaining substrates were 77 bp in length.

### Electromobility Shift Assays (EMSA)

Unlabeled or isotopically labeled substrates were incubated at room temperature for 1 h with increasing concentrations of His-VicR in 20 µl of binding buffer (25 mM Tris HCL pH 7.5, 12.5 mM KCl, 6.25 mM MgCl_2_, 10% glycerol). The reactions were separated on either 0.5 X TBE 8% or 12% native PAGE (29∶1 acrylamide: bisacrylamide). 8% PAGE gels were electrophoresed at 10 Volts/cm for 3–3.5 hrs at room temperature. 12% PAGE gels were also electrophoresed at 10 Volts/cm at room temperature for a length of 4.5 hrs. The radioactive gels were dried and exposed overnight with a phosphor screen (Fujifilm). Non-radioactive gels were stained in 1 X TBE with 1 X SYBR Green I Nucleic Acid Gel Stain (Invitrogen) for 25 minutes at room temperate with shaking. Both types of gels were visualized using a Typhoon FLA 7000 phosphorimager (GE Healthcare) and analyzed using ImageQuant version 5.0 (Molecular Dynamics).

### DNaseI Footprinting Analysis

Isotopically labeled substrates, with either the forward or reverse strand labeled, were incubated at room temperature with increasing concentrations of His-VicR (as shown in each figure) for 30 min in 50 µl of binding buffer (25 mM Tris HCl pH 7.5, 12.5 mM KCl, 6.25 mM MgCl_2_, 10% glycerol). The binding reactions were then subjected to DNase I digestion for 1 min by adding 0.25 U of DNaseI (Promega) in the presence of 2.5 mM CaCl_2_ and 5 mM MgCl_2_. The reactions were quenched by adding 90 µl of stop buffer (200 mM NaCl, 30 mM EDTA, 1% SDS, 100 µg/ml salmon sperm DNA) and subsequently purified by a phenol-chloroform extraction and overnight ethanol precipitation at −20°C. Samples were pelleted by centrifugation, dried and dissolved in sequencing sample buffer (95% formamide, 10 mM EDTA, 0.1% xylene cyanol, 0.1% bromophenol blue) and separated by electrophoresis with the appropriate sequencing reaction on a 6% polyacrylamide denaturing sequencing gel. Sequencing reactions for each gene were prepared using an unlabeled forward primer (except for *gtfB*) and the purified isotopically labeled fragment as a template with the SequiTherm EXCEL II DNA Sequencing Kit (Epicentre Biotechnologies). The sequencing gel was electrophoresed at 34 Volts/cm, dried, exposed to a phosphor screen (Fujifilm), scanned using a Typhoon FLA 7000 phosphorimager (GE Healthcare) and analyzed using ImageQuant version 5.0 (Molecular Dynamics).

### DNaseI footprinting with phosphorylated VicR

Footprinting reactions with phosphorylated VicR were carried out with 5 nM of the labeled *gcrR* substrate incubated at room temperature with increasing concentrations of His-VicR (phosphorylated and unphosphorylated control) as described above.

## Results

The Regulatory Sequence Analysis Tools (RSAT) Genome Scale DNA Pattern tool (http://rsat.ulb.ac.be/rsat) [27] was used to identify genes within the *S. mutans* UA159 genome that had matches to the *B. subtilis* WalR consensus. One substitution to the query sequence (TGTWAH-N_5_-TGTWAH) was permitted and was identified upstream of 274 genes. *gtfC, wapA, nlmC, gcrR* and *plsX* are a representative of those identified as having exact matches to the consensus. Representative genes with matches to the WalR consensus with one substitution were identified in the promoters of *atlA*, *gtfB*, *gbpB*, and *bmsH.* RSAT analysis did not yield matches to the consensus in *relP, glnQ* or *copY*, whose virulence traits have been demonstrated to be regulated by *vicRK* [9], [11], [28]. The upstream regions of these latter three genes were analyzed manually for potential matches to the WalR consensus because these genes were identified in a VicK mutant microarray (*copY)*, linked to competence (*relP*) or acid tolerance (*glnQ*) [9], [11], [29].

### VicR protection of targets harboring the WalR DNA binding consensus

**Figure 1:**
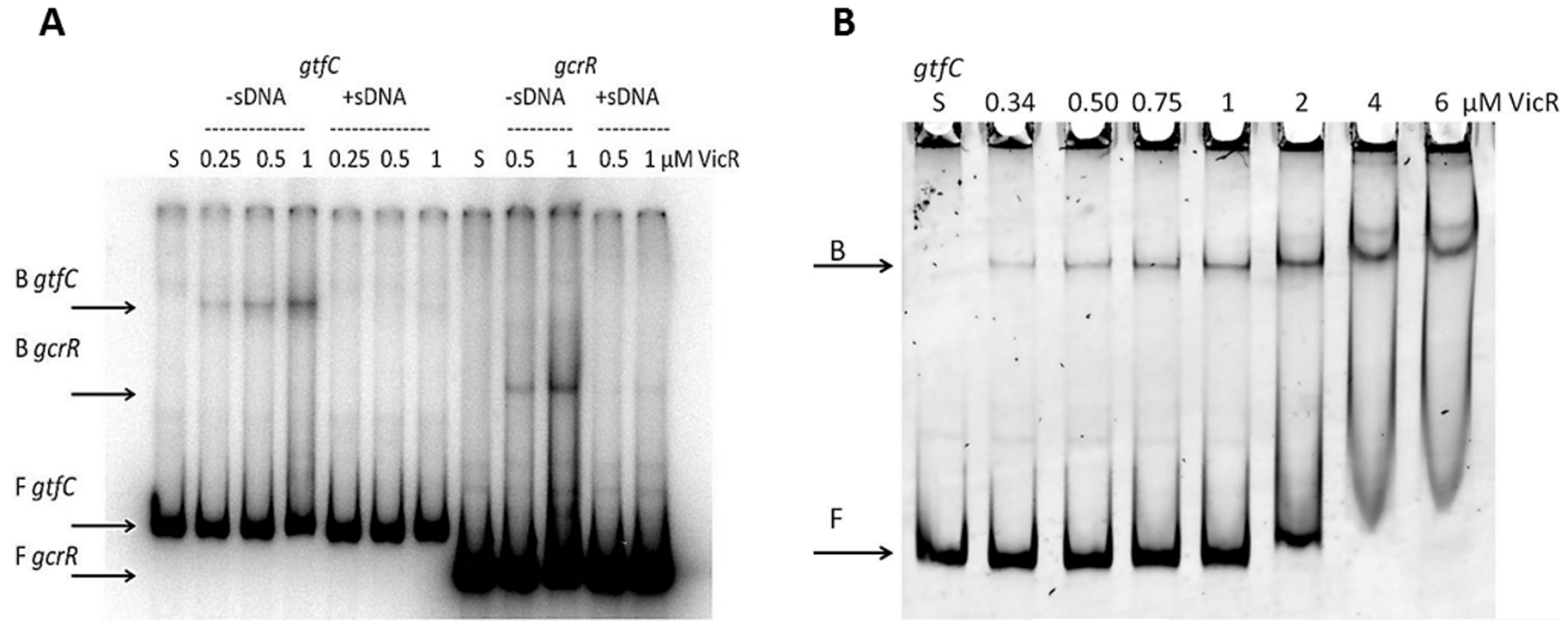
DNA binding assays of VicR to the *gtfC* and *gcrR* promoter regions. (A) EMSA analysis on the effect of salmon sperm DNA on VicR binding to the *gtfC* and *gcrR* promoter regions. Increasing concentrations of VicR was incubated with radiolabeled *gtfC* or *gcrR* probe with and without the addition of salmon sperm DNA. (B) EMSA analysis of a VicR titration with *gtfC* using SYBR Green staining. Increasing concentrations of VicR was incubated with labeled *gcrR* probe. The S (substrate only) above the first lane indicates that the *gtfC* substrate was incubated in the absence of VicR. F indicates free DNA and B indicates bound DNA.

We identified a perfect WalR consensus sequence upstream of a number of coding sequences within the *S. mutans* chromosome including *gcrR*, an orphan RR important in regulating the aciduricity of *S. mutans*. To determine if VicR binds to the promoter region of *gcrR*, EMSA was carried out on two isotopically labeled PCR substrates representing the *gtfC* and *gcrR* upstream regions. *gtfC* served as our positive control because it was identified as having a strong WalR consensus and had been previously shown to bind VicR [6]. Fig. 1A shows that His-VicR is able to bind both *gtfC* and *gcrR,* however only a weak interaction was observed. The addition of 100 µg/ml salmon sperm DNA (ssDNA) to the reaction greatly inhibited formation of the VicR-DNA substrate complex. We clearly show later in this study by DNaseI footprinting, that VicR indeed does specifically bind *gtfC* and *gcrR*.

To estimate the dissociation constant of VicR DNA binding, we carried out a VicR titration in the absence of ssDNA (Fig. 1B). EMSA was performed using unlabeled *gtfC* substrate and visualized by SYBR Green staining. We decided to switch from autoradiography to SYBR Green staining for EMSA because of the high background noise contributed by the free probe. From Fig. 1B we visually estimated the K_d_ to be between 2–4 µM.

**Figure 2:**
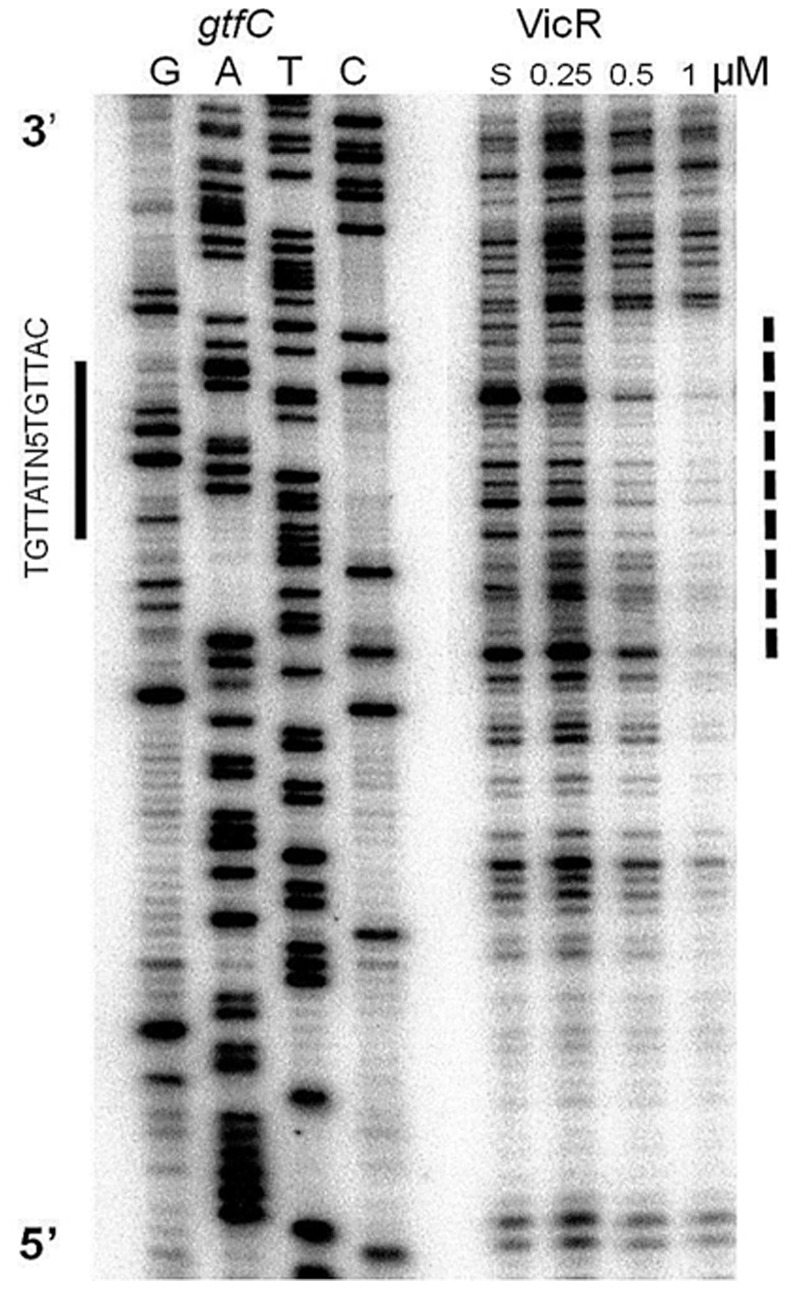
DNaseI footprint analysis of the *gtfC* promoter region. VicR was incubated with increasing concentrations of labeled *gtfC* probe. The S above the fifth lane indicates that the *gtfC* substrate was incubated in the absence of VicR. VicR protected a 33 bp region within the *gtfC* promoter (dashed line) that overlapped with the WalR consensus sequence (solid bar to the left of the autoradiograph). The lanes with the sequencing ladder as well as the lanes with the footprinting reactions are from the same gel with the intervening lanes removed.

The *gtfC* has a potential match to the WalR consensus (TGTTATagaagTGTTAC) 53 bp upstream of the start codon on the forward strand. The match to the consensus was proposed previously [6] but has not been confirmed by footprinting. The *gtfC* consensus overlaps with the promoter, where the first half-site of the putative binding site and the −35 region share six bases and the second half-site shares two bases with the −10 region (Fig. S5). To determine if the proposed VicR consensus sequence was accurate, we performed DNaseI footprinting by amplifying a 190 bp PCR product representing the region spanning the 157^th^ bp upstream of the start codon to the 30^th^ base downstream of the *gtfC* start codon.The isotopically labeled substrate was incubated with increasing amounts of purified His-VicR for 30 min., followed by DNaseI digestion. We detected a protected region covering 33 bp including the consensus and the *gtfC* promoter (Fig. 2). Our observations indicated that VicR bound to a direct repeat that matched the consensus sequence of its homologue WalR from *Bacillus* and *Staphylococcus*.

**Figure 3:**
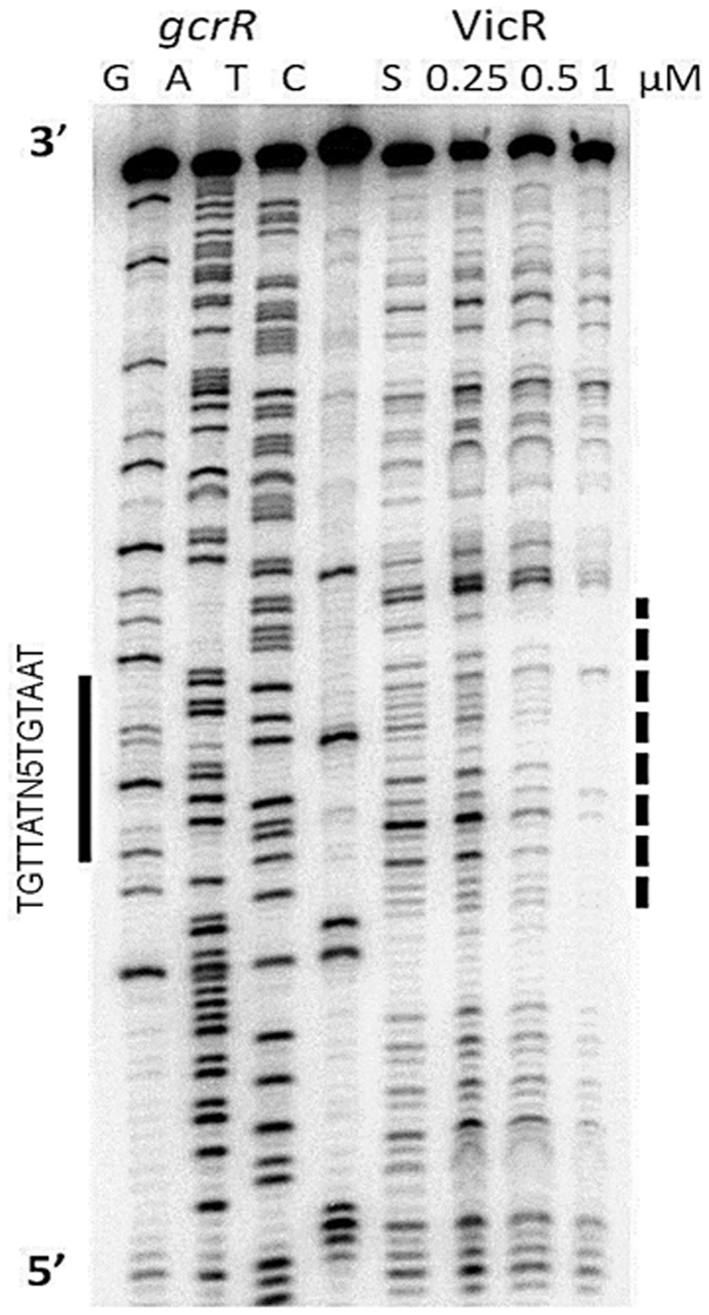
DNaseI footprint analysis of the *gcrR* promoter region. Labeled *gcrR* probe was incubated with no (indicated by the S above the fifth lane) or increasing amounts of VicR. The dashed line to the right of the autoradiograph indicates the region protected by VicR that overlapped the WalR consensus sequence (solid bar).

In *S. mutans*, GcrR (response regulator of the GbpC regulatory system) is an orphan RR that is involved in biofilm formation, sucrose mediated adhesion and the acid tolerance response [30]. GcrR was included in this study because it regulates virulence traits that overlap with the VicRK TCS and RSAT identified a perfect match to the WalR consensus binding site (TGTTATagaacTGTAAT) 94 bp upstream of the start codon on the forward strand (Fig. S5). To examine the VicR binding site, a 160 bp PCR product representing the region spanning the 181^st^ bp upstream of the start codon to the 23^rd^ base upstream of the *gcrR* start codon was footprinted. The VicR footprint indicated a protected region 22 bp in size located 110 bp downstream of the −10 site and 88 bp upstream of the start codon (Fig. 3). The protected region overlapped the WalR consensus sequence in the same manner that was observed for the *gtfC* promoter region.

**Figure 4:**
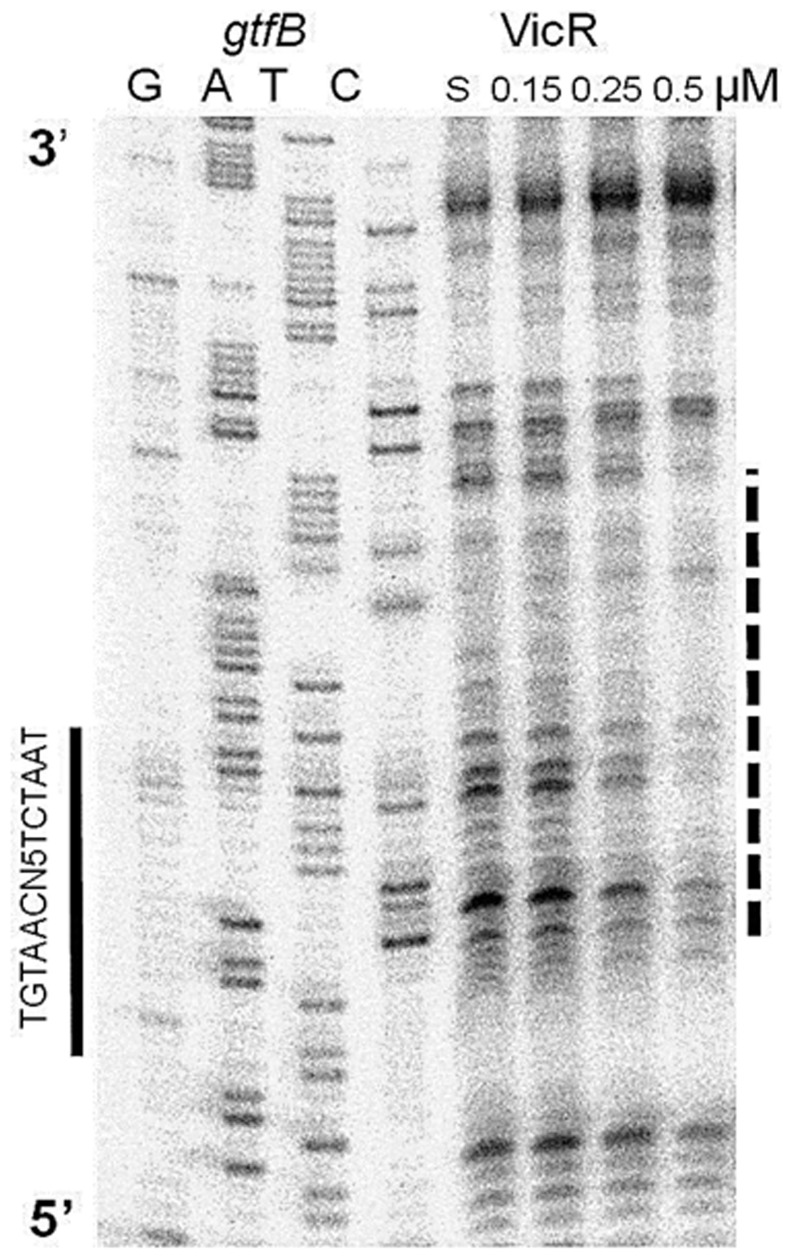
DNaseI footprint analysis of the *gtfB* promoter region. VicR at increasing concentrations was incubated with labeled *gtfB* probe. The S above the fifth lane indicates that the *gtfB* substrate was incubated in the absence of VicR. VicR protected a 35 bp region (dashed line) that partially overlapped the WalR consensus sequence (solid line).

After confirming that VicR bound to two perfectly matched WalR consensus sequences, we examined the binding site of VicR to an imperfect match of the WalR consensus. Using RSAT we identified a WalR consensus with one substitution upstream of *gtfB.* This sequence was located 46 bp upstream of the start codon on the reverse strand. This sequence differed from the consensus that was previously proposed by Senadheera et al., which is located 147 bp upstream of the start codon of *gtfB* [6]. To determine if VicR can bind to one or both of the proposed binding sites we performed DNaseI footprinting. A 195 bp PCR product representing the region spanning the 196^th^ bp upstream of the start codon to the second base upstream of the *gtfB* start codon was generated. This substrate contained both the previously proposed match to the WalR consensus and the new possible match to the consensus site identified by RSAT. Footprinting analysis revealed that VicR protected a 35 bp region that encompassed both the −10 and the −35 regions of the promoter and the newly identified putative binding site (TGTAACaccttTCTAAT) (Fig. 4). The protection was observed only within the last three bases (AAC) of the first half site, whereas the spacer and the second half site were completely protected. The *gtfB* region that was footprinted in this study was the same region that was used previously to show VicR binding by EMSA [6]. Our present studies explain why VicR was still able to bind despite the different binding sites that were proposed by Senadheera *et al.* [6], further demonstrating the importance of using sequence specific tools to identify actual VicR DNA binding sites. A negative control was included in our survey to exclude any non-specific binding of VicR. *fruA*, which encodes a fructan hydrolase, was used as a negative control because the promoter is well characterized and RSAT analysis revealed no matches to the WalR consensus [31]. A 240 bp substrate that represented a region that spans the 235^th^ bp upstream of the start codon to the 2^nd^ bp downstream of the *fruA* start codon was generated. Although no strong matches to the WalR consensus were identified, a weak match to the WalR consensus was identified manually with the following sequence, TGTAAGcgctaTCTTAT, 141 bp upstream of the start codon on the reverse strand. As shown in Fig. S1, there was no evidence of protection even with 2 µM VicR.

In addition to *gtfC*, *gcrR*, and *gtfB*, DNaseI footprinting was performed on several other DNA sequences that were identified by RSAT analysis as having a potential match to the WalR consensus (Fig. S2). A summary of the VicR DNA binding sequences are shown in Table 1. The genes that were analyzed in this study were grouped based on how strongly they were protected by VicR and whether their DNaseI footprints had defined boundaries (Table 1). For example the sites upstream of *gbpB*, *gcrR*, *gtfC*, *wapA*, *nlmC* and *bsmH* were all protected strongly by VicR (Fig. S2 and [11]) and were listed at the top of Table 1 (+++). Strong protection in this study was defined as the absence of visible DNaseI cleavage products in a delineated region of a lane on a sequencing gel with 0.5 µM VicR. In contrast, *glnQ* gave a defined footprint (Fig. S2) but only at a high (2.0 µM) VicR concentration and was listed at the bottom of Table 1 (+).

**Table 1:** Summary of the VicR consensus sequences as determined by DNaseI footprinting.

Gene	Consensus Match (TGTWAH_N5_TGTWAH)	Strand	Affinity
*gbpB*	TGTAAT_N5_CGTAAT	F	+++
*gcrR*	TGTTAT_N5_TGTAAT	F	+++
*gtfC*	TGTTAT_N5_TGTTAC	F	+++
*wapA*	TGTTAT_N5_TGTTAT	R	+++
*nlmC*	TGTAAA_N5_TGTTAA	F	+++
*bmsH*	TATTAA_N5_TGTTAT	F	+++
*gtfB*	TGTAAC_N5_TCTAAT	R	++
*plsX*	TGTTAT_N5_TGTAAC	F	++
*copY*	TATAAC_N15_TGTCAA	R	++
*atlA*	TCTAAT_N5_TGTTAT	F	+
*relP*	TCTTAC_N5_TGTTCT	R	+
*glnQ*	TGTTAG_N17_TGTTAT	R	+

**Figure 5:**
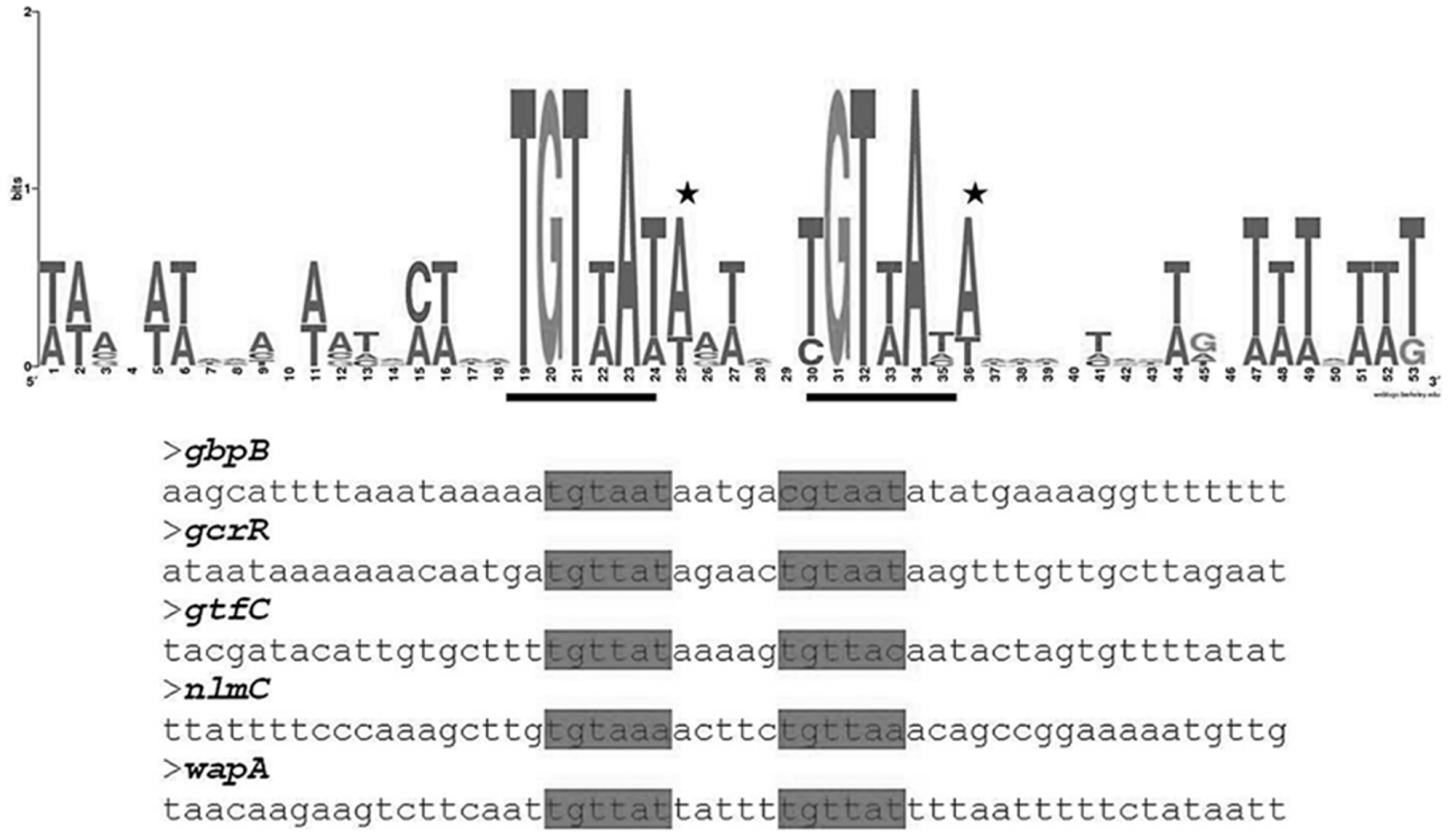
WebLogo alignment of high affinity VicR targets with a 5 base pair spacer. The output of the WebLogo software is featured above. The hexameric half sites are indicated by the black bars. The stars indicate the conserved adenines immediately following the half sites. The sequences that were used for the sequence alignment are below the Weblogo. The hexameric sites are highlighted within the sequences.

*gbpB, gcrR, gtfC, wapA* and *nlmC* all contained a good match to the *B. subtilis* WalR consensus and for this study were designated as strong binding sites for *S. mutans* VicR. The protected regions from these genes were used to generate a WebLogo [32] sequence alignment pattern to search for bases beyond the WalR consensus that may be specific to VicR binding (Fig. 5). Fig. 5 shows that there are very strongly conserved nucleotides beyond the hexameric half-sites of the WalR consensus. The half-sites as well as the adenines that immediately follow them were mutagenized (see below).

### Mutagenesis of the WalR consensus in *S. mutans*

We carried out a mutational analysis to dissect the WalR consensus in *S. mutans* and determine the components that are essential for recognition by VicR. *gcrR* was chosen for mutagenesis because the gene product plays a key role in virulence, has a perfect RSAT match to the *B. subtilis* WalR consensus and has a relatively high affinity for VicR as observed by footprinting (Fig. 3). We also sought to determine if the bases immediately following each half site played a role in recognition by VicR, and to investigate this theory further we also mutated the binding site upstream of *plsX*. Like *gcrR*, the *plsX* site has a good match to the *B. subtilis* WalR consensus but differs from the high affinity binders in the bases that immediately follow the hexameric half sites, making it an ideal candidate to look for subtle changes in binding strength due to bases outside of the consensus.

The *gcrR* match (TGTTATagaacTGTAAT) to the *B. subtilis* WalR consensus consists of two hexameric direct repeat (DR) half sites (referred to in this study as DRI and DRII respectively) separated by a five bp spacer. A 77 bp substrate comprising the WalR consensus direct repeats upstream of *gcrR* was generated as described in the Materials and Methods. The sequence of each mutated DR was based on our approximation of the least frequently found base within each individual WalR consensus half site of *gtfC, wapA, nlmC* and *gcrR*. DRI was substituted with GACGGC and DRII was substituted with GACGGG but the spacer was left unmodified.

**Figure 6:**
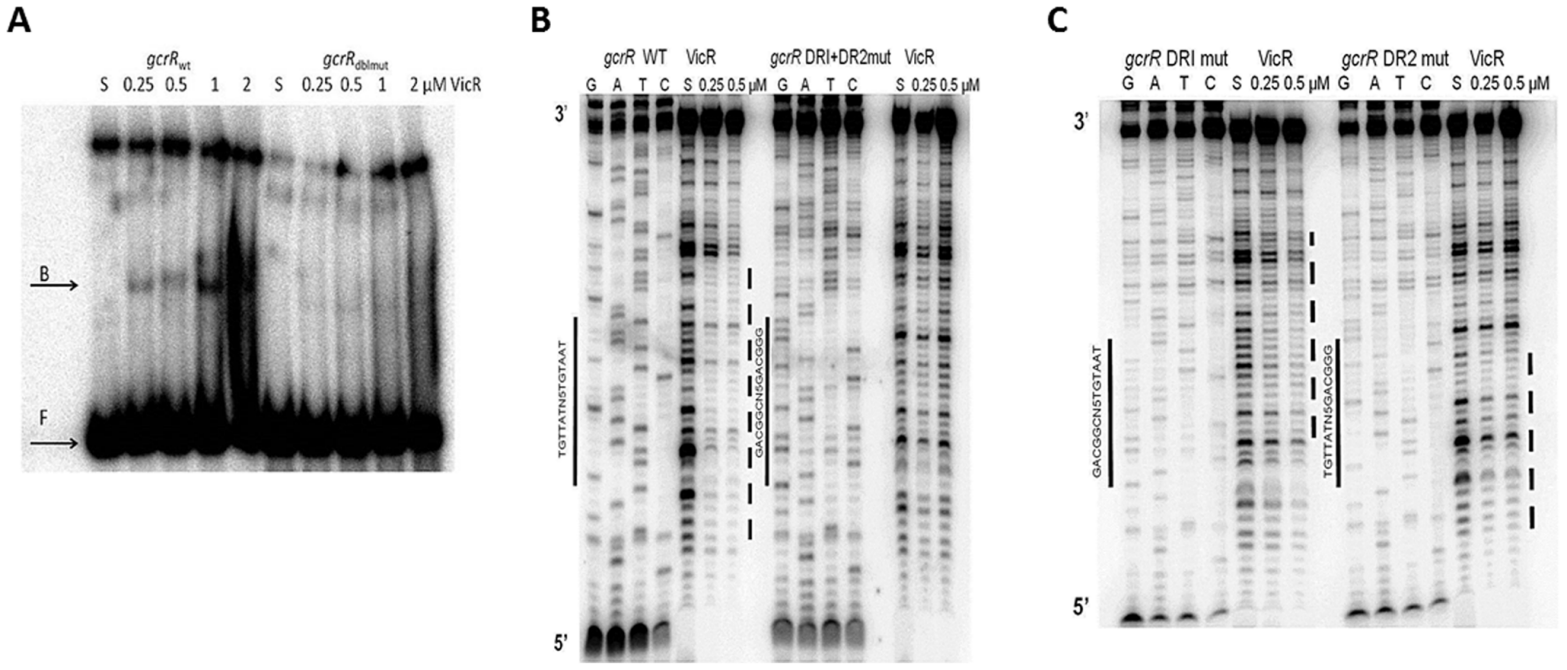
DNA binding analysis of VicR with wildtype and mutated promoter regions of *gcrR*. (A) EMSA analysis of *gcrR* 77 bp variants. VicR at increasing concentrations was incubated with labeled wildtype and mutated *gcrR* probe. The S above the fifth lane indicates that the *gcrR* substrate was incubated in the absence of VicR. F indicates free DNA, B indicates bound DNA. (B) DNaseI footprint analysis of a *gcrR* 77 bp wildtype (WalR consensus TGTTATagaacTGTAAT) and a *gcrR* double DR mutant (WalR mutated consensus GACGGC agaacGACGGG). VicR at increasing concentrations was incubated with labeled probe. The S above the fifth lane indicates that the substrate was incubated in the absence of VicR. The solid bars indicate the match to the WalR consensus and the mutated sequence. The dashed line indicates the region protected by VicR. (C) DNaseI footprint analysis of individual *gcrR* DR mutants. VicR at increasing concentrations was incubated with labeled probe. The S above the fifth lane indicates that the substrate was incubated in the absence of VicR. The solid bars indicate the match to the WalR consensus and the mutated sequences. The dashed lines indicate the regions protected by VicR.

Mutation of both the *gcrR* DRI and DRII eliminated binding as observed by EMSA (Fig. 6A) and DNaseI footprinting (Fig. 6B) of otherwise identical 77 bp DNA substrates. This observation shows that the *B. subtilis* WalR consensus accurately predicts a binding site for VicR in *S. mutans*. Individual mutations of each DR demonstrated that each half site contributes to VicR binding; however the effect of each DR mutation was not equal. The wild type 77 bp substrate was strongly protected across a 33 bp region that covered both DRs as well the 8 bp upstream of DRI and the 8 bp downstream of DRII (Fig. 6B). In the DRI mutant, the protection was limited to a region 21 bp wide and showed strong protection of the entire spacer, DRII, and the 8 bp downstream of this half-site (Fig. 6C). The DRII mutant showed protection of a region 26 bp wide that encompassed the 8 bp upstream of DRI, DRI, the spacer and the first base of the mutant DRII sequence. However the protection across the footprint of the DRII mutant was much weaker when compared to that of either the wild type or the DRI mutant substrate (Fig. 6C).

**Figure 7:**
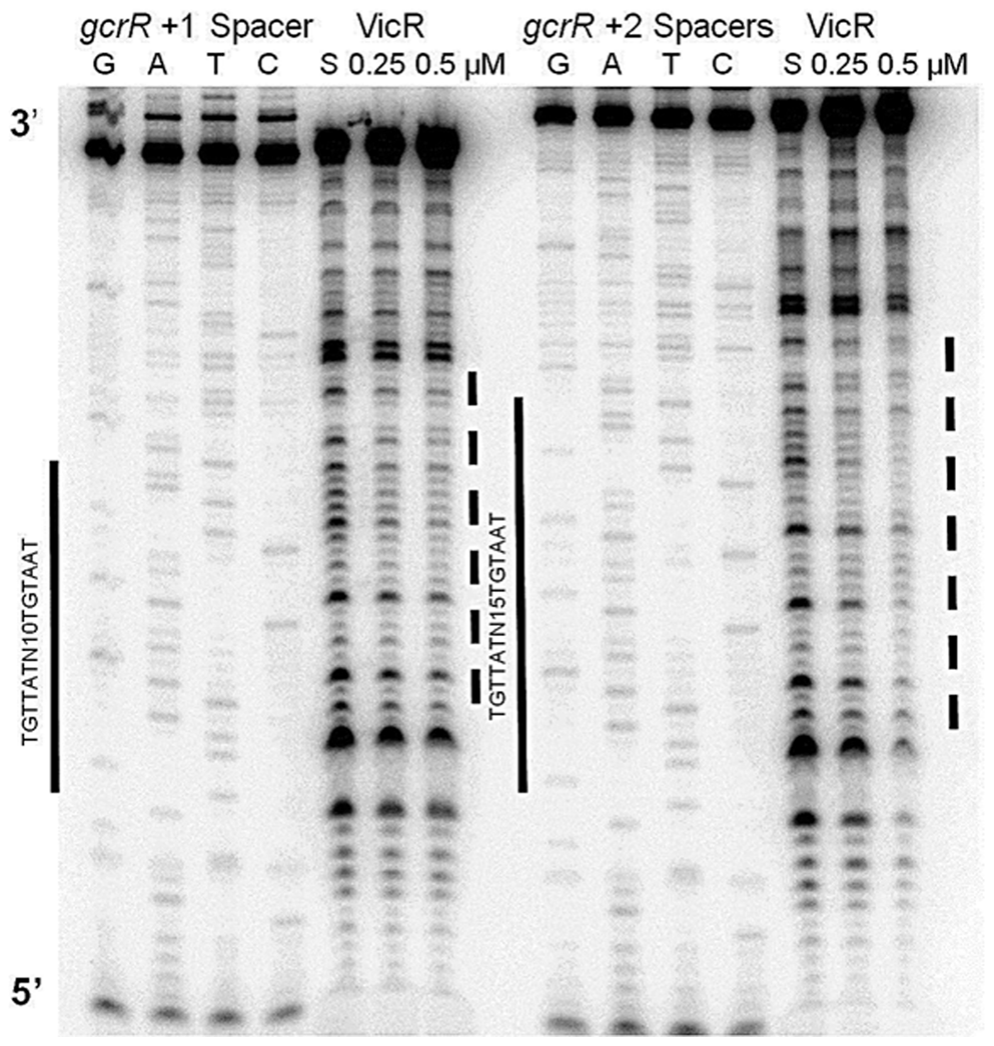
DNaseI footprint analysis of *gcrR* helical phasing mutants. Labeled probe was incubated with no (S above the fifth lane) or increasing amounts of VicR. The solid bars indicate the match to the WalR consensus and the mutated sequences. The dashed lines indicate the regions protected by VicR. The +1 spacer is a 82 bp substrate and the +2 spacer is a 87 bp substrate.

To test if the length of the spacer between the direct repeats affected recognition of the consensus by VicR, the length of the spacer region was increased by doubling (+1 spacer) and tripling (+2 spacer) the existing sequence. DRI and DRII were unmodified in these mutants. Assuming a helical turn for B-form DNA to be 10.6 bp, the extra 5 bp between the DRs in the +1 spacer mutant placed each DR on opposite sides of the helix with respect to one another, whereas an extra 10 bp, as seen in the +2 spacer mutant, brings both direct repeats back in phase. The +1 spacer mutant showed diminished binding, but protection was not eliminated. The +2 spacer also showed diminished binding but was closer to the protection seen with the wild type sequence (Fig. 7).

**Figure 8:**
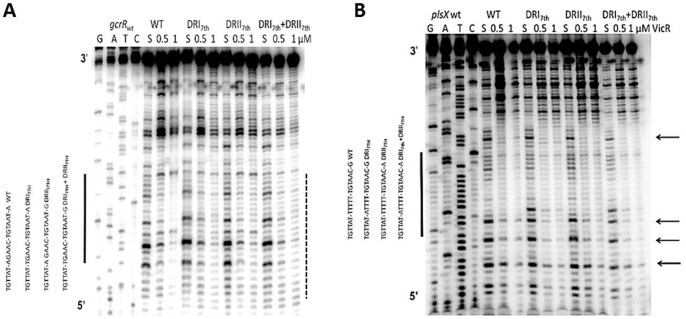
DNaseI footprinting assays of VicR with the 7^th^ position mutants of *gcrR* and *plsX* promoter regions. (A) DNaseI footprint analysis of *gcrR* 7^th^ position mutants. VicR at increasing concentrations was incubated with labeled probe. (B) DNaseI footprint analysis of *plsX* 77 bp 7^th^ position mutants. VicR at increasing concentrations was incubated with labeled probe. The S indicates that the substrate was incubated in the absence of VicR. The solid bars to the left of the autoradiograph indicate the match to the WalR consensus. The arrows indicate the bases that are referentially protected in the 7^th^ position mutants.

Based on the conservation of the adenines following each DR in genes that exhibited strong protection and in light of the helical phasing results above, we decided to test our hypothesis that the bases immediately following the consensus contribute to the strength of binding of VicR. Since we have shown that DRI and DRII appear to be the strongest determinants for VicR binding we predicted that the bases immediately following each DR would have a subtle effect on binding. As such, we chose two targets that also showed a subtle difference in binding by VicR. VicR displayed a strong affinity towards *gcrR* and less affinity for *plsX* as judged by the degree of protection to DNase I (Fig. 8A and 8B). Both showed a very similar composition with regards to their DRs, differing only in the last base of DRII. On the other hand both sites differ in the composition of the spacer as well as the bases following each DR. The bases comprising the 7^th^ position immediately downstream of each DR (designated as DRI_7_ and DRII_7_ respectively) were mutated to investigate the roles they play in regards to VicR binding. *gcrR* was used as a positive control because it contains an adenosine in the 7^th^ position in both DRI_7th_ and DRII_7th_. *plsX* was our negative control as it contains a thymidine in the DRI_7th_ and a guanosine in the DRII_7th_. The *gcrR* seventh 7^th^ position mutants were made to resemble *plsX*. In turn, the *plsX* 7^th^ position mutants were made to resemble *gcrR*. We predicted that the *gcrR* mutants would show a decrease in VicR protection while the *plsX* mutants would show an improvement. As expected the individual *gcrR* DRI_7th_ and DRII_7th_ mutants and the double 7^th^ position mutant showed visibly diminished binding with respect to the wild type *gcrR* sequence at 1 µM VicR (Fig. 8A).

Three *plsX* mutants were generated with mutations in DRI_7th_, DRII_7th_ and both DRI_7th_+ DRII_7th_ (double mutant). Each of these mutants was made to resemble *gcrR* at the indicated seventh position. Each *plsX* mutant was preferentially protected at four bases (indicated by arrows in Fig. 8B) compared to the wild type *plsX* sequence. We observed an improvement in VicR binding with each of the single mutants and a cumulative effect with the double mutant (Fig. 8B).

### The Effect of VicR Phosphorylation on DNA binding activity

We revisited the weak interaction that we observed in regards to VicR binding to *gcrR* by EMSA (Fig. 1A). Phosphorylation of RRs can increase the affinity of a RR for its target. Although phosphotransfer from *S. mutans* VicK to VicR has been shown [33], VicK also comprises phosphatase activity [17]. We were concerned that the phosphatase activity of VicK would dephosphorylate VicR before the DNA binding event could occur [17]. Therefore, we chose to carry out our DNaseI footprinting reactions with VicR phosphorylated with acetyl phosphate instead of VicK. Acetyl phosphate was used for this study because it is readily available, inexpensive and was recently shown to phosphorylate *Streptococcus pneumoniae* VicR *in vitro* [17]. *S. mutans* VicR was incubated in phosphorylation buffer with or without acetyl phosphate. VicR appears to be phosphorylated to completion as only the slower migrating higher molecular weight band was present in lane 2 of Fig. S3. We found that by pre-chilling and running the gels at 4°C we could resolve the unphosphorylated and phosphorylated states of VicR in the same lane. A tightly spaced doublet representing these two forms can be seen in lane 3 of Fig. S3.

After seeing evidence of acetyl phosphate mediated VicR phosphorylation (Fig. S3) we proceeded to test the ability of phosphorylated VicR to bind and protect DNA by DNaseI footprinting. We expected a change in the affinity of phosphorylated VicR over VicR with regard to the target binding site. Unphosphorylated and phosphorylated VicR at 0.25 µM and 0.5 µM were allowed to incubate for 1 h at room temperature with [γ-^32^P] labeled *gcrR* substrate. Both sets of reactions protected a region that surrounds the target sequence (TGTTATagaacTGTAAT) (Fig. S4). There was no significant change in protection of the substrate in the presence or absence of acetyl phosphate as determined by the size and intensity of the footprint (Fig. S4).

## Discussion

In the present study, EMSA was carried out to confirm the ability of His-VicR to bind a known member of the VicR regulon. We found that while our His-VicR was able to form a stable complex with the upstream region of *gtfC*, this interaction was easily disrupted by the addition of non-specific competitor DNA (Fig. 1A). We estimated the K_d_ to be 2–4 µM based on the EMSA analysis using SYBR Green (Fig. 1B). Half B_max_ (available binding sites), an estimate of K_d_ is approached at 2 µM VicR and most of the probe is shifted as the concentration is increased to 4 µM. This K_d_ is high for a RR considering that the K_d_ calculated by analytical ultracentrifugation for each half site in *E. coli* KdpE, another OmpR family member, is 350 and 200 nM respectively [34]. Our EMSA results are in line with binding studies done with *S. mutans* His-VicR by Stipp *et al*., as they show that His-VicR is able to shift several targets (*wapE*, *lysM*, *smaA* and *smu.367*) between 1.2–4.8 µM [23].

Weak interactions detected by EMSA may be a characteristic of proteins related to VicR. The sporulation RR, Spo0A, has structural homology to the DNA binding domain of the OmpR family of RRs [1]. Direct binding of Spo0A to members of its regulon has been shown by EMSA but only in the absence of competitor DNA [35]. Spo0A requires the assistance of RNA polymerase to bind target DNA when salmon sperm DNA is present in the EMSA loading buffer [36]. Spo0A like VicR, demonstrates weaker DNA binding affinity, but analysis of binding is readily accomplished by DNaseI footprinting [1], [37].

For each gene that was footprinted in this study, we compared the locations of the *B. subtilis* WalR consensus, footprint, promoter and start codon (Fig. S5). The promoter sequences of *gbpB*, *nlmC*, *copY*, *glnQ*, *gtfB*, *gtfC*, *wapA*, and *gcrR* were obtained from the literature [11], [38]–[43] and BPROM, an online bacterial promoter prediction tool (www.softberry.com/berry.html) was used to predict the location of the *bmsH*, *relP* and *plsX* promoters. DNaseI footprinting analysis revealed that VicR binding overlapped the WalR consensus in all genes tested (Fig. S2).

Matches to the WalR consensus were identified on either the coding or non-coding strand of genes in this study manually or using RSAT. The predicted location of the WalR consensus on a particular strand of the bacterial chromosome did not determine the affinity of VicR binding. *nlmC*, *gtfC*, *wapA*, *gbpB* and *gcrR*, which were the best substrates for VicR, contain consensus on opposite strands, but demonstrated relatively high affinity footprints that tightly correlated with the consensus.

Based on RT-PCR and microarray data VicR negatively regulates *nlmC* and *copY* [11] and positively regulates *atlA*, *gbpB*, *bmsH*, *gtfB*, and *gtfC* [6], [9], [10] indicating that VicR is a RR capable of repressing and activating transcription. The effect of VicR on the transcriptional regulation of *gcrR*, *glnQ, plsX*, *relP* and *wapA* has not been investigated.

Transcriptional activators often bind upstream or within the promoter itself [44]. DNA binding proteins can bind to direct repeats and have been shown to act as both activators and repressors of targets [1] where positively regulated genes contain binding sites that are upstream of the −35 sequence of the promoter and negatively regulated genes contain sites that overlap the promoters and extend into the +1 transcriptional start site. In *B. subtilis,* Spo0A regulation also depends on the location of the binding site relative to the promoter [1]. Genes regulated by WalR in *B. subtilis* also cluster coincidentally with the location of the consensus [45]. Unfortunately, for VicR we cannot draw a clear correlation between the consensus site relative to the location of the promoter and a particular mode of transcriptional regulation as seen with Spo0A. For example both *copY* and *gtfC*, which have opposite modes of regulation, have promoters that overlap with the footprint and the consensus. *bmsH* has a consensus downstream of the promoter but, unlike genes regulated by SpoA, it is positively regulated. The location of the consensus on a distinct strand of a gene also does not predict transcriptional regulation by VicR. The VicR consensus sequence of *nlmC*, *bmsH*, *gtfC*, *gbpB*, *gcrR* and *plsX* are located on the coding strand of each gene. In contrast, the consensuses for *gtfB*, *wapA*, *atlaA*, *relP*, *glnQ*, and *copY* are found on the non-coding strand. *bsmH* and *atlA* are both positively regulated by VicR but have a consensus that is found on opposite strands. Likewise *nlmC* and *copY* are negatively regulated but also have their consensus sequences on different strands. Finally *nlmC* and *gtfC*, which are oppositely regulated, both have their consensus sequences on the coding strand.

The consensus sequence that dictates high affinity binding of *S. mutans* VicR appears to be similar for *B. subtilis* WalR. Mutation of either DR inhibited binding, while a double DR mutant completely eliminated protection of the VicR binding site. Spacer mutations were created to determine if the spacing between the DRs was important and also to conclude if cooperativity of VicR was occurring at each DR. The duplication of the spacer region of the VicR binding site also diminished binding of VicR but did not eliminate it. There are several possibilities for the results seen with the spacer repeat mutants. The first explanation is that VicR is able to bind to the individual half sites. The second explanation is that the spacer may contain bases that are contacted specifically by VicR, thus stabilizing the interaction with the DNA substrate. As seen from the WebLogo results (Fig. 5), the *gcrR* spacer does appear to contain at least one highly conserved base immediately following DRI that may play a role in VicR binding. Lastly, adding repeats of the spacer may have inadvertently created a new VicR DR. The sequence TGTCTT and TGTTCT are introduced in the double spacer and triple spacer mutants. While not perfect matches to the DRs of the WalR consensus (TGTWAH), they are close approximations, which may have artificially increased the number of contacts for VicR to recognize. This might have had a stabilizing effect on the binding reaction despite the DRs being out of phase. This is somewhat different from the mutational analysis done on the *B. subtilis* WalR binding site, where mutation of the first three bases of a single half-site or the addition of a base pair to the spacer abolished binding of WalR to the *yocH* promoter [21].

It is not surprising that VicR, a protein so well conserved and essential across so many Gram-positive species, also shares conservation of the highest affinity sequence determinants. However there is room for additional bases to provide increased strength of binding that lie outside the conserved consensus. We observed this in the 7^th^ position of each DR for *gcrR* and *plsX*. We argued that the bases at this position could potentially dictate subtle differences in VicR binding strength. We found that by mutating the bases in the 7^th^ position we could selectively induce a small change in the preference for a site by VicR.

The adenosines found in the 7^th^ position of the high affinity VicR targets might be widely conserved as they are also found immediately following the equivalent half-sites in the *E. coli* PhoB consensus [46]. PhoB makes contact with the sigma subunit of the RNA polymerase holoenzyme with the alpha loop [46]. A similar mode of transcriptional activation may be true for VicR since the PhoB consensus, CTGTCATA-T/A-A-T/A-CTGTCACA-T/A-T/A-N [46] is very similar to the *B. subtilis* WalR consensus. Here we propose modification to the WalR consensus for *S. mutans*. Based on the WebLogo alignment (Fig. 5) and mutation of the bases in the 7^th^ positions of the WalR consensus (Fig. 8), the *S. mutans* VicR consensus appears to be TGTWAHA-N_4_-TGTWAHA. This reflects the conservation of the essential DRs from the WalR consensus as well as the additional bases that we discovered here.

The modified consensus, TGTWAHA-N_4_-TGTWAHA was used in an RSAT search of the *S. mutans* UA159 genome. *gtfC*, *nlmC* and *gcrR* were identified by this sequence when no substitutions to the modified consensus were allowed. This is to be expected as these were three of the genes used to generate the WebLogo [32]. Using RSAT, when one substitution of the modified consensus was allowed, *gbpB*, *gtfB*, *nlmA*, and *wapE* were among the genes identified all of which contain the conserved adenines following each direct repeat. In contrast, RSAT did not identify a VicR consensus sequence upstream of *copY*, *relP*, and *glnQ*; genes that display virulence phenotypes that are *vicRK* dependent [9], [11], [28]; however a VicR consensus was observed when these promoter regions were searched manually. One reason RSAT did not identify consensus sequences upstream of *copY* and *glnQ* is the presence of 10 additional bp (or one helical turn) within the spacer region between direct repeats. Although these genes do not comprise an exact VicR consensus sequence, DNaseI footprinting revealed binding of VicR upstream of these genes at sites which overlapped the predicted consensus sequence (Table 1). Furthermore, these genes contain adenines in the 7^th^ position of the direct repeat (with the exception of the first direct repeat of *glnQ*) which could further assist in allowing recognition by VicR.

We found that *S. mutans* VicR, like *S. pneumoniae* VicR, can be phosphorylated by acetyl phosphate (Fig. S3). Acetyl phosphate has been shown to be an effective phosphoryl donor to RRs *in* *vitro* [47] and has also been shown to be an important, though not essential, phosphoryl donor to RRs *in vivo*. We observed a reduction in VicR migration rates in response to phosphorylation with a gradient SDS-PAGE gel that had been pre-chilled and electrophoresed at 4°C (Fig. S3). Phosphorylation-induced migration rates by SDS-PAGE analysis has been shown in other proteins, including the mammalian heart protein Phospholambam, type II cAMP-dependent protein kinase, and glycogen synthase kinase [48]. In addition, phosphorylation of the transcription factor CREB binding protein (CBP) and Tau protein causes a reduction in the rate of migration compared to their unphosphorylated forms [49] [50]. The reduction in mobility may be due to differences in binding of SDS by proteins [51], and may also be due to conformational changes that are retained even after treatment with SDS [48].

We hypothesized that phosphorylation of VicR would result in an alteration of binding affinity for its DNA targets since it belongs to the OmpR family of RRs. Phosphorylation of OmpR and PhoB induced homodimerization followed by enhanced DNA binding [52], [53]. OmpR binding to the high affinity site upstream of *ompF* was dramatically enhanced by phosphorylation with acetyl phosphate. The K_d_ for the F1 site drops dramatically from 151 nM to 6 nM upon phosphorylation [54]. In contrast to OmpR, there was no evidence that phosphorylation played a role in increasing the affinity of VicR for the binding site upstream of *gcrR*. We performed a DNaseI footprinting assay on the promoter region of *gcrR*, a known target of VicR, with unphosphorylated and phosphorylated VicR but we found no alternation in the protected region (Fig. S4). Although it’s possible that a small portion of the phosphorylated VicR converts to the unphosphorylated form during the course of our binding experiments, we think that the stability of the phosphorylated form throughout SDS PAGE indicates that the phosphorylated form is sufficiently stable to have demonstrated any affinity differences during the course of our EMSA and footprinting experiments.

Studies with CovR, another OmpR family member, from *S. pyogenes* show that phosphorylation can lead to conformational changes that affect oligomerization in addition to DNA binding [55]. Footprinting analysis shows that wild type CovR has a two-fold increase in affinity for the *has* promoter when it is phosphorylated [55]. However, phosphorylation does not always result in a change in affinity for a target sequence. In contrast to the studies above our lab has shown that phosphorylation of *S. mutans* ComE likely plays a role in oligomerization instead of promoting DNA binding [56].

In this study we show for the first time proof of direct binding of *atlA*, *bmsH*, *glnQ*, *copY*, *wapA*, *relR*, *gcrR* and *plsX by S. mutans* VicR. We show that binding is mediated by the WalR consensus. We also determined the essential components of the WalR consensus in *S. mutans* as well as bases beyond it that aid in recognition by VicR. We demonstrate that VicR is capable of using acetyl phosphate as a phosphodonor *in vitro* and under these conditions phosphorylation has no effect on the affinity of this RR for its DNA target. The research in this study provides some insight into how this essential RR recognizes target virulence genes. Beyond the contribution to the general study of DNA-protein interactions the pursuit of the details of the mechanism of VicR target recognition is important to biomedicine. The biochemistry of the VicRK TCS presents a promising target for the development of new antimicrobial therapies.

## Supporting Information

Figure S1 **DNaseI footprint analysis of the** ***fruA*** **promoter region.** VicR at increasing concentrations was incubated with labeled *fruA* probe. The S above the fifth lane indicates that the *fruA* substrate was incubated in the absence of VicR.
(DOCX)

Figure S2 **DNaseI footprint analysis of the** ***gbpB*** **(A),** ***wapA*** **(B),** ***bmsH*** **(C),** ***copY*** **(D),** ***atlA*** **(E),** ***relP*** **(F),** ***glnQ*** **(G) promoter regions.** VicR at increasing concentrations was incubated with labeled DNA substrates. The S above the fifth lane indicates that the DNA substrate was incubated in the absence of VicR.
(DOCX)

Figure S3 **Phosphorylation of VicR with acetyl phosphate.** Lane 1: VicR; Lane 2: VicR plus acetyl phosphate (VicR+P); Lane 3∶1∶1 mixture of VicR plus VicR+P.
(DOCX)

Figure S4 **DNaseI footprint of** ***gcrR*** **with phosphorylated VicR.** Lane 1: Substrate only; Lane 2∶0.25 µM VicR; Lane 3∶0.5 µM VicR; Lane 4∶0.25 µM VicR plus acetyl phosphate; Lane 5∶0.5 µM VicR plus acetyl phosphate. The footprint boundary is indicated by the dashed line.
(DOCX)

Figure S5 **Relative locations of the VicR footprint, the promoter, and the consensus from genes used in this study.** The dashed line represents the region footprinted by VicR. The boxes labeled −35 and −10 represent the promoter half sites. The boxes labeled DRI and DRII represent WalR consensus half sites. The long solid arrow represents the forward strand of each gene. Consensus boxes above this arrow are found on the coding strand of each gene. Boxes below the arrow are found on the non-coding strand. Transcriptional regulation by VicR of each gene is indicated by PR (positive regulation), NR (negative regulation) and UR (unknown regulation).
(DOCX)

Table S1 **Primers used to generate substrates for footprinting and EMSA.**
(DOCX)

Table S2 ***gcrR*** **mutational analysis primers.**
(DOCX)

Table S3 ***plsX*** **mutational analysis primers.**
(DOCX)
