# Supplementary figures and images for: A Biochemical Characterization of the DNA Binding Activity of the Response Regulator VicR from Streptococcus mutans
Source: PLoS One. 2014 Sep 17;9(9):e108027. doi: 10.1371/journal.pone.0108027 (PMC4168254; doi:10.1371/journal.pone.0108027)

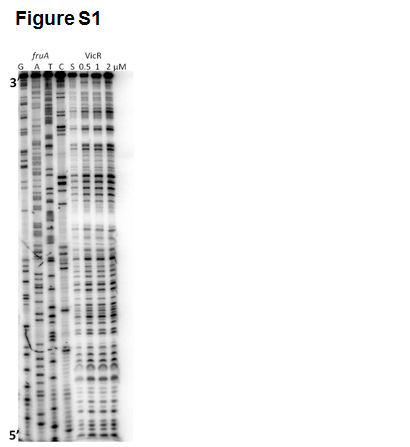

Supplement: Figure S1 — DNaseI footprint analysis of the fruA promoter region. VicR at increasing concentrations was incubated with labeled fruA probe. The S above the fifth lane indicates that the fruA substrate was incubated in the absence of VicR. (DOCX) [file pone.0108027.s001.docx]

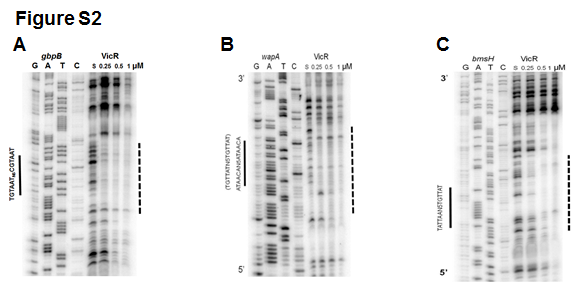


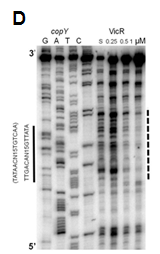

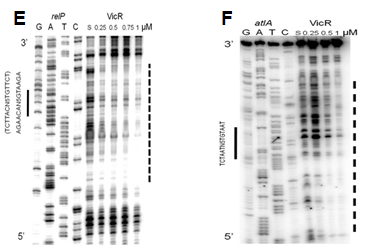

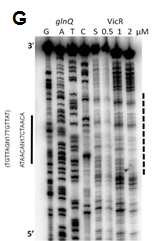

Supplement: Figure S2 — DNaseI footprint analysis of the gbpB (A), wapA (B), bmsH (C), copY (D), atlA (E), relP (F), glnQ (G) promoter regions. VicR at increasing concentrations was incubated with labeled DNA substrates. The S above the fifth lane indicates that the DNA substrate was incubated in the absence of VicR. (DOCX) [file pone.0108027.s002.docx]

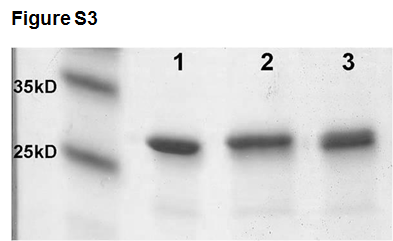

Supplement: Figure S3 — Phosphorylation of VicR with acetyl phosphate. Lane 1: VicR; Lane 2: VicR plus acetyl phosphate (VicR+P); Lane 3∶1∶1 mixture of VicR plus VicR+P. (DOCX) [file pone.0108027.s003.docx]

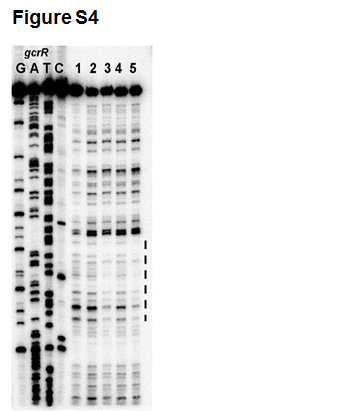

Supplement: Figure S4 — DNaseI footprint of gcrR with phosphorylated VicR. Lane 1: Substrate only; Lane 2∶0.25 µM VicR; Lane 3∶0.5 µM VicR; Lane 4∶0.25 µM VicR plus acetyl phosphate; Lane 5∶0.5 µM VicR plus acetyl phosphate. The footprint boundary is indicated by the dashed line. (DOCX) [file pone.0108027.s004.docx]

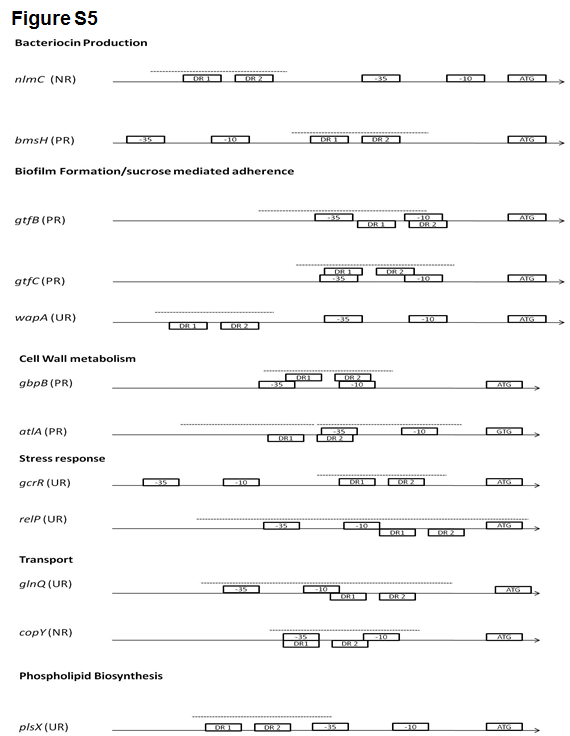

Supplement: Figure S5 — Relative locations of the VicR footprint, the promoter, and the consensus from genes used in this study. The dashed line represents the region footprinted by VicR. The boxes labeled −35 and −10 represent the promoter half sites. The boxes labeled DRI and DRII represent WalR consensus half sites. The long solid arrow represents the forward strand of each gene. Consensus boxes above this arrow are found on the coding strand of each gene. Boxes below the arrow are found on the non-coding strand. Transcriptional regulation by VicR of each gene is indicated by PR (positive regulation), NR (negative regulation) and UR (unknown regulation). (DOCX) [file pone.0108027.s005.docx]
