# Supplementary material for: A Biochemical Characterization of the DNA Binding Activity of the Response Regulator VicR from Streptococcus mutans
Source: PLoS One. 2014 Sep 17;9(9):e108027. doi: 10.1371/journal.pone.0108027 (PMC4168254; doi:10.1371/journal.pone.0108027)
Supplement: Table S1 — Primers used to generate substrates for footprinting and EMSA. (DOCX) [file pone.0108027.s006.docx]

**Table S1.** Primers used to generate substrates for footprinting and EMSA

| Primer | Sequence  5’ to 3’ | Gene target |
| --- | --- | --- |
| oSG491  oSG242  oSG584  oSG585  oSG588  oSG589  oSG674  oSG675  oSG813  oSG814  oSG582  oSG583  oSG736  oSG737  oSG534  oSG535  oSG753  oSG754  oSG755  oSG756  oSG578  oSG579  oSG809  oSG810  oSG621  oSG622  oSG580  oSG581 | gcgggatccaatgaagaaaattc  cccaagcttgctaataaaattcgtaaaaataagggac  ttacaattatgaggttattc  actgactgttttttattcac  ataaaattcaagagcttcta  caaaatggatgaaatgagga  gtgaagaggcagaaactaag  catttgaaatagatgtcatt  gacatctgtcatattaagga  tccatttccttgctctcctt  tttctcttctttcaaaaact  tcttttcattacaaatataa  gattttaatagattttgttt  caatttctataatatttctt  tggcaaatgaaaaccaacaa  ccacatttttaaaggtaatgagtgc  caattagactgttgtttttttgg  taggaacctccaaattttaaactg  tctagggttaggagttttaa  tttacgtaatttaaaacgta  gaataataaatccagccttg  gactgatgaattagagatta  gatatgctaaaataattgat  cttcataatccaactcctcg  aaaacgatgccttgctgact  atgctctaaaatctgagaca  taaaaccttcaaaacaaaat  tagttatatctacatctaca | VicR  VicR  *atlA*  *atlA*  *bsmH*  *bsmH*  *copY*  *copY*  *fruA*  *fruA*  *gbpB*  *gbpB*  *gcrR*  *gcrR*  *glnQ*  *glnQ*  *gtfB*  *gtfB*  *gtfC*  *gtfC*  *nlmC*  *nlmC*  *plsX*  *plsX*  *relR*  *relR*  *wapA*  *wapA* |
