# Supplementary material for: A Biochemical Characterization of the DNA Binding Activity of the Response Regulator VicR from Streptococcus mutans
Source: PLoS One. 2014 Sep 17;9(9):e108027. doi: 10.1371/journal.pone.0108027 (PMC4168254; doi:10.1371/journal.pone.0108027)
Supplement: Table S2 — gcrR mutational analysis primers. (DOCX) [file pone.0108027.s007.docx]

**Table S2.** *gcrR* mutational analysis primers

| Primer | Sequence  5’ to 3’ | Gene target |
| --- | --- | --- |
| oSG712  oSG713  oSG710  oSG711  oSG716  oSG717  oSG718  oSG719  oSG720  oSG721  oSG722  oSG723  oSG724  oSG725  oSG841  oSG842  oSG843  oSG844  oSG845  oSG846  oSG867  oSG868  oSG863  oSG864  oSG869  oSG870 | tttcccattataataataaa  atcaaactataaattctaag  tttcccattataataataaaaaaacaatgatgttatagaactgtaataag  atcaaactataaattctaagcaacaaacttattacagttctataacatca  tttcccattataataataaaaaaacaatgaGACGGCagaactgtaataag  atcaaactataaattctaagcaacaaacttattacagttctGCCGTCtca  tttcccattataataataaaaaaacaatgatgttatagaacGACGGGaag  atcaaactataaattctaagcaacaaacttCCCGTCgttctataacatca  tttcccattataataataaaaaaacaatgaGACGGCagaacGACGGGaag  atcaaactataaattctaagcaacaaacttCCCGTCgttctGCCGTCtca  tttcccattataataataaaaaaacaatgatgttatagaacagaactgtaataag  atcaaactataaattctaagcaacaaacttattacagttctgttctataacatca  tttcccattataataataaaaaaacaatgatgttatagaacagaacagaactgtaataag  atcaaactataaattctaagcaacaaacttattacagttctgttctgttctataacatca  tttcccattataataataaaGGGGTGGCAGtgttatagaactgtaataag  atcaaactataaattctaagcaacaaacttattacagttctataacaCTG  tttcccattataataataaaaaaacaatgatgttatGAGGTtgtaataag  atcaaactataaattctaagcaacaaacttattacaACCTCataacatca  tttcccattataataataaaaaaacaatgatgttatagaactgtaatGGT  atcaaactataaattctaagTGGTCCCACCattacagttctataacatca  tttcccattataataataaaaaaacaatgatgttattgaactgtaataag  atcaaactataaattctaagcaacaaacttattacagttcaataacatca  tttcccattataataataaaaaaacaatgatgttatagaactgtaatGag  atcaaactataaattctaagcaacaaactCattacagttctataacatca  tttcccattataataataaaaaaacaatgatgttattgaactgtaatgag  atcaaactataaattctaagcaacaaactcattacagttcaataacatca | *gcrR*  *gcrR*  *gcrR*  *gcrR*  *gcrR*  *gcrR*  *gcrR*  *gcrR*  *gcrR*  *gcrR*  *gcrR*  *gcrR*  *gcrR*  *gcrR*  *gcrR*  *gcrR*  *gcrR*  *gcrR*  *gcrR*  *gcrR*  *gcrR*  *gcrR*  *gcrR*  *gcrR*  *gcrR*  *gcrR* |
