# Supplementary material for: A Biochemical Characterization of the DNA Binding Activity of the Response Regulator VicR from Streptococcus mutans
Source: PLoS One. 2014 Sep 17;9(9):e108027. doi: 10.1371/journal.pone.0108027 (PMC4168254; doi:10.1371/journal.pone.0108027)
Supplement: Table S3 — plsX mutational analysis primers. (DOCX) [file pone.0108027.s008.docx]

**Table S3.** *plsX* mutational analysis primers

| Primer | Sequence  5’ to 3’ | Gene target |
| --- | --- | --- |
| oSG826  oSG827  oSG818  oSG819  oSG824  oSG825  oSG847  oSG848  oSG849  oSG850  oSG851  oSG852 | agtagccttaattttgctga  aattataccatgaataaaag  agtagccttaattttgctgaattgtttatttgttatttttttgtaacgtt  aattataccatgaataaaagaatcttaaacgttacaaaaaaataacaaat  agtagccttaattttgctgaattgtttatttgttatagaactgtaatgtt  aattataccatgaataaaagaatcttaaacattacagttctataacaaat  agtagccttaattttgctgaattgtttatttgttatAtttttgtaacgtt  aattataccatgaataaaagaatcttaaacgttacaaaaaTataacaaat  agtagccttaattttgctgaattgtttatttgttatttttttgtaacAtt  aattataccatgaataaaagaatcttaaaTgttacaaaaaaataacaaat  agtagccttaattttgctgaattgtttatttgttatAtttttgtaacAtt  aattataccatgaataaaagaatcttaaaTgttacaaaaaTataacaaat | *plsX*  *plsX*  *plsX*  *plsX*  *plsX*  *plsX*  *plsX*  *plsX*  *plsX*  *plsX*  *plsX*  *plsX* |
